# RepID represses megakaryocytic differentiation by recruiting CRL4A-JARID1A at DAB2 promoter

**DOI:** 10.21203/rs.3.rs-3045396/v1

**Published:** 2023-06-26

**Authors:** Jae-Hyun Jo, Seon-Mi Ok, Dong-Kyu Kim, Yeong-Mu Kim, Jong-Uk Park, Dong-Hyun Jung, Hye-Ji Kim, Hyun-A Seong, Hyo Je Cho, Jihoon Nah, Sangjune Kim, Haiqing Fu, Christophe E. Redon, Mirit I. Aladjem, Sang-Min Jang

**Affiliations:** Chungbuk National University; Chungbuk National University; Chungbuk National University; Chungbuk National University; Chungbuk National University; Chungbuk National University; Chungbuk National University; Chungbuk National University; Chungbuk National University; Chungbuk National University; Chungbuk National University; NCI, NIH; NCI, NIH; NCI, NIH; Chungbuk National University

**Keywords:** Megakaryocytic differentiation, RepID, CRL4A, JARID1A

## Abstract

**Background:**

Megakaryocytes (MKs) are platelet precursors, which arise from hematopoietic stem cells (HSCs). While MK lineage commitment and differentiation are accompanied by changes in gene expression, many factors that modulate megakaryopoiesis remain to be uncovered. Replication origin binding protein (RepID) which has multiple histone-code reader including bromodomain, cryptic Tudor domain and WD40 domains and Cullin 4-RING ubiquitin ligase complex (CRL4) recruited to chromatin mediated by RepID have potential roles in gene expression changes via epigenetic regulations. We aimed to investigate whether RepID-CRL4 participates in transcriptional changes required for MK differentiation.

**Methods:**

The PCR array was performed using cDNAs derived from RepID-proficient or RepID-deficient K562 erythroleukemia cell lines. Correlation between RepID and DAB2 expression was examined in the Cancer Cell Line Encyclopedia (CCLE) through the CellMinerCDB portal. The acceleration of MK differentiation in RepID-deficient K562 cells was determined by estimating cell sizes as well as counting multinucleated cells known as MK phenotypes, and by qRT-PCR analysis to validate transcripts of MK markers using phorbol 12-myristate 13-acetate (PMA)-mediated MK differentiation condition. Interaction between CRL4 and histone methylation modifying enzymes were investigated using BioGRID database, immunoprecipitation and proximity ligation assay. Alterations of expression and chromatin binding affinities of RepID, CRL4 and histone methylation modifying enzymes were investigated using subcellular fractionation followed by immunoblotting. RepID-CRL4-JARID1A-based epigenetic changes on DAB2 promoter were analyzed by chromatin-immunoprecipitation and qPCR analysis.

**Results:**

RepID-deficient K562 cells highly expressing MK markers showed accelerated MKs differentiation exhibiting increases in cell size, lobulated nuclei together with reaching maximum levels of MK marker expression earlier than RepID-proficient K562 cells. Recovery of WD40 domain-containing RepID constructs in RepID-deficient background repressed DAB2 expression. CRL4A formed complex with histone H3K4 demethylase JARID1A in soluble nucleus and loaded to the DAB2 promoter in a RepID-dependent manner during proliferation condition. RepID, CRL4A, and JARID1A were dissociated from the chromatin during MK differentiation, leading to euchromatinization of the DAB2 promoter.

**Conclusion:**

This study uncovered a role for the RepID-CRL4A-JARID1A pathway in the regulation of gene expression for MK differentiation, which can form the basis for the new therapeutic approaches to induce platelet production.

## Background

Megakaryocytes (MK) are derived from hematopoietic stem cells (HSCs) via a differentiation process called megakaryocytic differentiation and are responsible for the production of blood platelets (1, 2). MKs-derived platelets play a major role in thrombosis, inflammation, hemostasis and wound healing (3). During megakaryocytic differentiation, MKs undergo an increase in cytoplasm size, polyploidization, and nuclear reduplication, followed by a maturation step called thrombopoiesis that generates platelet (4, 5). Besides morphological alterations, gene expression is also dynamically changed. High-throughput studies, including microarray or whole-genome and RNA sequencing during K562 erythroleukemia progenitor cells and pluripotent hematopoietic stem cells differentiation to MKs identified several genes such as Integrin subunit aIIb (ITGA2B;CD41), Integrin subunit b3 (ITGB3;CD61) and Disabled homolog 2 (DAB2) that are highly correlated with megakaryocytic differentiation (6–14). Still, the underlying mechanism and regulatory factors involved in MK differentiation are largely unknown.

The replication origin binding protein RepID, also known as DDB1/CUL4-associated factor 14 (DCAF14) or pleckstrin homology domain-interacting protein (PHIP), exhibits pleiotropic functions, including replication fork stability (15), replication origin initiation (16, 17), mitosis (18) and metastasis (19). Mutations in RepID are linked to dysmorphic features intellectual disability, developmental delay, behavioral problems and hypotonia (20). While most DCAFs are regarded as substrate recognition modules for Cullin-RING anchored ubiquitin ligases, RepID is a structural DCAF that recruits the CUL4-anchored ubiquitin machinery (hereafter Cullin 4-RING E3 ubiquitin ligase or CRL4) onto chromatin. Once recruited to chromatin, CRL4 can initiate the degradation of chromatin components such as the chromatin licensing and DNA replication factor 1 (CDT1) or the Budding Uninhibited by Benzimidazoles 3 (BUB3) (16, 18). RepID-mediated CRL4 chromatin loading is achieved the bromo, cryptic tudor, and WD40 functional domains of RepID, which are crucial for the recognition of modified histones (17, 21–27). CRL4 also interacts with core components of histone methylation complexes, including EED, Polycomb-group protein, and the core subunit WDR5 of the human MLL histone H3K4 methyltransferase that are essential for epigenetic and transcription controls. Therefore, CRL4 inactivation impairs histone modifications (28). The Cullin-4B anchored CRL4B can promote chromatin silencing through its association with the polycomb-repressive complex PRC2, the histone methyltransferase SUV39H1, the heterochromatin protein 1 (HP1) and the DNA methyltransferase 3A (DNMT3A), leading to H2AK119 monoubiquitination and subsequently to H3K27 trimethylation, H3K9 trimethylation and DNA methylation (29, 30). These molecular characteristics suggest that RepID and CRL4 are crucial epigenetic controllers that trigger gene transcription or silencing, but the mechanistic details of those interactions remain to be fully elucidated.

Here, we report that RepID represses MK differentiation by recruiting CRL4A to the DAB2 promoter, resulting in heterochromatin formation. RepID dissociates from chromatin in response to megakaryocytic differentiation signals, thereby preventing the loading of CRL4A as well as the histone H3K4 demethylase JARID1A, a novel interaction partner of CRL4A, to the DAB2 promoter. These molecular events correlated with increased transcription of megakaryocyte markers and megakaryocytic morphological changes in a subpopulation of RepID-depleted cells. We also observed that megakaryocytic differentiation is accelerated in RepID-depleted cells upon stimulation by phorbol 12-myristate 13-acetate (PMA). These findings provide an example of gene transcription regulation during megakaryocytic differentiation with the RepID-dependent recruitment of an epigenetic modifier.

## Methods

### Cell culture and chemicals

Human K562 and DMS114 cells, parental and RepID knockout, were cultured in Dulbecco’s modified Eagle’s medium (Invitrogen, 10569–010) or RPMI-1640 medium (Invitrogen, 11875–093) supplemented with 10% heat-inactivated fetal bovine serum in a 37°C/5% CO2 humidified incubator (16, 18). All original cell lines were obtained from American Type Culture Collection (ATCC) (www.atcc.org) and tested for mycoplasma (Lonza, LT07–418). Cycloheximide, MG132, and PMA were purchased from Sigma (Cat. C4859), Millipore (Cat. 11134515001), and Santa Cruz (Cat. Sc-3576), respectively. Each compound was added to the medium at the indicated concentrations.

### Immunofluorescence analysis

Slides of proliferating or differentiating K562 cells were prepared using TXT3 Cytospin (Nasco Korea Corp., Seoul, Korea) at 900×*g* for 5 min. Slides were rinsed in phosphate-buffered saline (PBS; pH 7.4), fixed with 4.0% paraformaldehyde (PFA) for 10 min, and permeabilized using PBS-T buffer [0.1 % Triton X-100 in 1×PBS, phenylmethylsulfonyl fluoride (PMSF), phosphatase inhibitor cocktail (Roche, P4906845001), and protease inhibitor cocktail (Sigma, P8340)] for 10 min on ice. Primary antibody staining was performed using anti-DAB2 (Cell Signaling, 12906, 1:1000). Secondary antibody staining was performed using Alexa Fluor^™^ Plus 488 conjugated anti-rabbit IgG (Invitrogen, A32731, 1:500). DNA was counterstained with DAPI (Thermofisher Scientific, 62248). A Zeiss LSM880 confocal microscope was used for imaging.

### ChIP-qPCR analysis

Chromatin immunoprecipitation (ChIP) analyses were performed on 1% formaldehyde-fixed proliferating or differentiating K562 cells using a Pierce^™^ magnetic ChIP assay kit (Thermofisher Scientific, 26157). Antibodies used in this study included normal rabbit IgG (Cell Signaling, 2729, 1:500), anti-CUL4A (Abcam, ab92554, 1:100), anti-JARID1A (Abcam, ab78322, 1:100), anti-H3K4me3 (Millipore, 05–1339, 1:100) and anti-H3K9me3 (Millipore, 05–1250, 1:100). Immunoprecipitated DNA and control input DNA was analyzed by quantitative real-time PCR using the human DAB2 promoter-specific primers; forward:5’-CTC GCG GAG CTC AGG GGA G-3,’ reverse:5’-GGT AAC TCC CCC TCA ACG TG-3. ‘

### RT2-profiler PCR array and qRT-PCR analysis

Proliferating K562 RepID WT and KO cells were harvested, and RNA was extracted using the RNeasy R Mini Kit (Qiagen, 74104) and converted to cDNA by using PrimeScript RT Master Mix (Takara, RR036A) according to the manufacturer’s instructions. An RT^2^ Profiler PCR array (Qiagen, PAMM-043Z) with 84 genes associated with the Wnt signaling pathway was used.

Total RNA extraction, cDNA synthesis, and qRT-PCR were performed according to the manufacturer’s instructions provided by Qiagen (RNeasy R Mini Kit, 74104), Takara (PrimeScript RT Master Mix RR036A), and Invitrogen (SYBR Green PCR Master Mix 4309155), respectively. The expression levels of human DAB2, CD41, and CD61 in the cells were determined by qRT-PCR, using GAPDH as an internal control. DAB2 primers; forward:5’-CCA GAT GCA AGA GGG GAT AA-3’ and reverse:5’-TCC TCC ACA CAC GTA ACC AA-3,’ CD41 primers; forward:5’-AGG TGA GAG GGA GCA GAA CA-3’ and reverse:5’-TCC ACC TTG AGA GGG TTG AC-3,’ CD61 primers; forward:5’-GAC AAG GGC TCT GGA GAC AG-3’ and reverse:5’-ACT GGT GAG CTT TCG CAT CT-3,’ GAPDH primers; forward:5’-GAG TCA ACG GAT TTG GTC GT-3’ and reverse:5’-TTG ATT TTG GAG GGA TCT CG-3.’

### Subcellular fractionation, co-IP, and immunoblotting

Cytosol, membrane, soluble nucleus, and chromatin fractions from proliferating or PMA-mediated differentiating K562 cells were prepared using a subcellular protein fractionation kit (Thermofisher Scientific, 78840) together with PMSF, protease inhibitor cocktail, and phosphatase inhibitor cocktail. Cell lysis was performed according to the manufacturer’s protocol. Total cell lysates were immunoprecipitated using an anti-CUL4A (Abcam, ab92554), or anti-JARIDIA (Abcam, ab78322) at 3 μg of antibody per sample. After rotation 14 h at 4°C, 60 μl of Sepharose beads (Millipore, 11134515001) were added and samples were incubated for an additional 2 h at 4°C in rotator. Protein-antibody-bead complexes were collected by centrifugation at 2000×*g* for 3 min and washed three times with PBS. Sixty μl 2×SDS Laemmli sample buffer (Bio-Rad, 1610737) was added, and the complexes were boiled for 15 min. Protein binding or each subcellular fraction was immunodetected following SDS-PAGE. Unprocessed original blots are shown in the Supplementary file.

The following primary antibodies were used: anti-DAB2 (Cell Signaling, 12906, 1:1000), anti-CUL4A (Abcam, ab92554, 1:20,000), anti-CUL4B (Sigma, C9995, 1:1000), anti-RepID (Abcam, ab86244, 1:2000), anti-DDB1 (Cell Signaling, 5428, 1:1000), anti-JARID1A (Cell Signaling, 3876, 1:1000; Abcam, ab78322, 1:2000), anti-WDR5 (Cell Signaling, 13105, 1:1000), anti-RBBP5 (Cell Signaling, 13171, 1:1000), anti-ASH2L (Cell Signaling, 5019, 1:2000), anti-FLAG (Sigma, F1804, 1:2000), anti-histone H3 (Millipore, 07–690, 1:25,000), and anti-a-tubulin (Sigma, T9026, 1:10,000). For secondary antibodies, horseradish peroxidase (HRP)-conjugated anti-mouse IgG (Cell Signaling, 7076) and HRP-conjugated anti-rabbit IgG (Cell Signaling, 7074) were used following the manufacturer’s suggested protocols.

### Proximity ligation assay (PLA)

The Duolink in situ Red starter mouse/rabbit PLA fluorescence assay (Sigma, DUO92101) was performed according to the manufacturer’s instructions. Briefly, proliferating or PMA-induced differentiating K562 cells were attached to slides using cytospin. Cells were washed with 1×PBS and fixed for 15 min at 4°C in 4% paraformaldehyde in PBS and permeabilized with 0.1% Triton X-100 in PBS for 5 min at 4°C. The coverslips were blocked with Duolink blocking solution and incubated with CUL4A (Abcam, ab92554, 1:200) and JARID1A (Abcam, ab78322, 1:200) antibodies in Duolink antibody diluent overnight, followed by incubation with PLUS and MINUS PLA probes, ligation, and amplification. The coverslips were then washed and mounted using mounting medium containing DAPI. Images were captured using a wide-field microscope, processed, and analyzed using Zeiss Zen 3.7 and ImageJ software.

### Cell morphology analysis

Proliferating or differentiating K562 cells were attached to slides using TXT3 cytospin (Nasco Korea) at 900×*g* for 5 min and stained with Wright-Giemsa staining solution (Sigma, 45253 and 32884). Cell morphology, including cell size and polynucleation, was analyzed by microscopy and ImageJ software. At least 290 cells in randomly separated fields were counted and expressed as relative cell size or percentage of the total cell number in these fields.

### Database analysis

Pearson’s correlation R values according to the gene expression levels between CRL4 components (RepID, CUL4A, CUL4B, and DDB1) and histone H3K4 methyltransferase/demethylase were analyzed using the Cancer Cell Line Encyclopedia (CCLE) dataset from the CellMiner cross-database (CellMinerCDB, https://discover.nci.nih.gov/cellminercdb/)(31). The Pearson correlation R-value was represented as a heatmap. Interaction profiles between CRL4 components and histone H3K4 methylation modifiers were obtained from the Human BioGRID Interaction Database (version 4.4.214 Oct 2022).

## Results

### RepID negatively correlates with DAB2 expression

**Figure 1:**
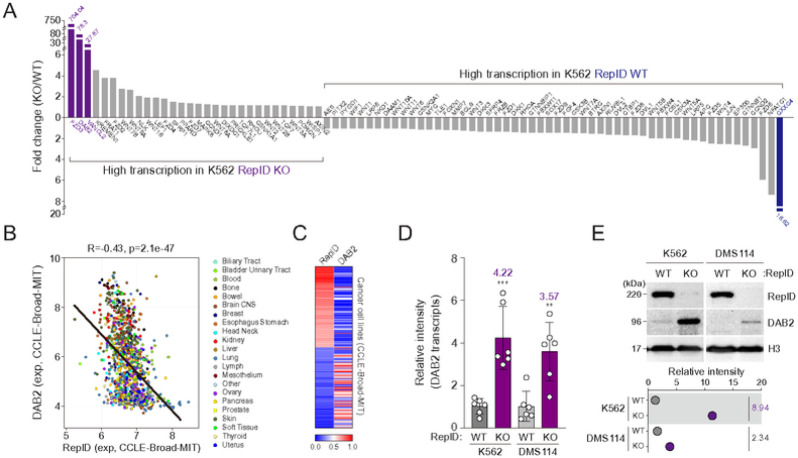
RepID negatively correlates with the DAB2 expression (A) Profiling of transcription levels of genes involved in Wnt signaling pathway in wild-type (WT) or RepID-depleted (KO) K562 cancer cell lines using PCR array. (B, C) DAB2 transcription is negatively correlated with the RepID. Expression of DAB2 transcripts was compared with RepID transcripts in all cancer dataset based on Cancer Cell Line Encyclopedia (CCLE) database using CellMinerCDB (B). Heatmap of transcription correlation between RepID and DAB2 from CCLE dataset (C). (D) DAB2 transcription is increased in a RepID-depleted cancer cell lines. Transcription level of DAB2 was examined by qRT-PCR in K562 or DMS114 cell lines in RepID WT or RepID-depleted condition. Average of transcription level relative to GAPDH was indicated, and error bars represent the standard deviation (**P-value < 0.01, ***p < 0.001). (E) DAB2 protein is highly expressed in RepID-deficient cancer cell lines. Immunoblot analysis with antibodies against DAB2 was performed and Histone H3 was used as loading control. Relative intensity of DAB2 protein was indicated as fold changes of RepID KO to RepID WT cells.

To determine whether RepID participates in transcriptional changes, we performed a PCR array assay using cDNAs derived from RepID-proficient or RepID-deficient K562 erythroleukemia cell lines. Interestingly, the highest observed gene deregulation involved the Wnt/b-catenin pathway with Frizzled 3 receptor (FZD3), Disabled-2 (DAB2), and Van Gogh-like 2 (VANGL2) being upregulated in RepID-deficient K562 cells more than 20 fold (Fig. 1A, left panel). Conversely, CXXC Finger Protein 4 (CXXC4) increased 16.62 folds in RepID WT K562 cells (Fig. 1A, right panel). Interestingly, the Wnt signaling has been shown to have a profound role in platelet production (32). Of interest, DAB2 was shown to be upregulated when both hematopoietic stem cells and the human K562 cells undergo megakaryocytic differentiation (33). In silico analyses of cancer cell lines from the Cancer Cell Line Encyclopedia (CCLE) via the Cellminer database showed that the expression of RepID transcripts negatively correlates with DAB2 expression (Fig. 1B, C). Subsequent quantitative reverse transcription PCR and immunoblot analysis at a subset of cancer cell lines, such as DMS114 or K562 cells, induced transcription as well as protein expression levels of DAB2 in RepID-depleted conditions (Fig. 1D, E). Altogether, our results imply that RepID may transcriptionally repress DAB2 expression in some cancer cells.

### RepID-deficient K562 accelerates megakaryocytic differentiation

**Figure 2:**
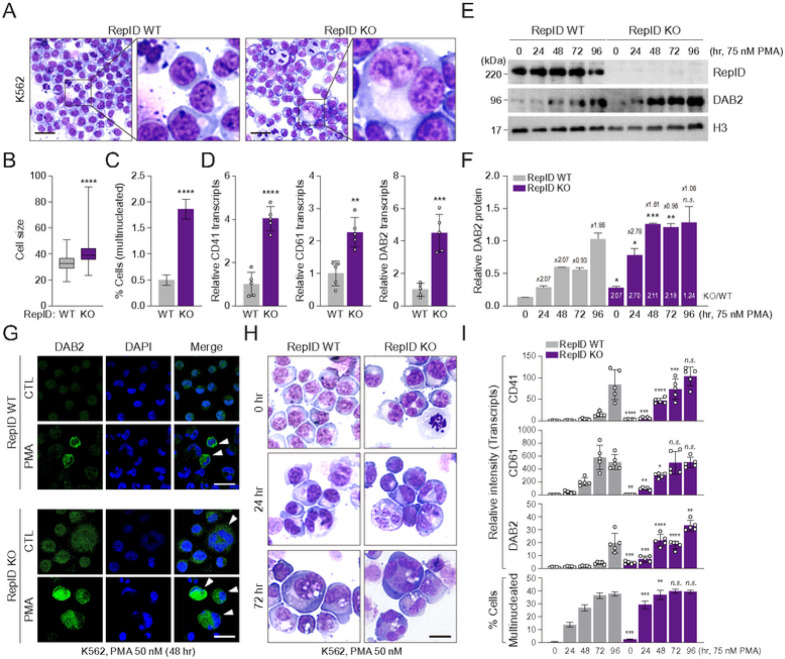
RepID depletion accelerates megakaryocytic differentiation (A-D) Subpopulations of RepID-deficient K562 cells show megakaryocytic phenotype. Representative images of Wright-Giemsa stained RepID WT and KO K562 cells (A). scale bar: 50 mm. Cell size of RepID WT (n=329) and KO (n=290) K562 cells was shown in (B). Percentage of multinucleated cells (WT: n = 2367, KO: n = 3803) (C). Transcription levels of megakaryocyte marker including CD41, CD61 and DAB2 in K562 RepID WT and KO cells (D). (E-G) Expression level of DAB2 protein is highly induced in RepID-deficient K562 cells by PMA treatment. Immunoblot analysis with antibodies against DAB2 was performed using PMA-treated K562 RepID WT and KO cells, and histone H3 was used as loading control (E). The numbers above the bar graph represent the fold increase of DAB2 protein level at the indicated time after PMA treatment compared to 24 hr before, and the numbers in the bar graph represent the DAB2 protein expression in RepID KO cells versus RepID WT cells (F). Immunofluorescence analysis of DAB2 expression in CTL or PMA-treated K562 RepID WT or KO cells. White arrows indicate DAB2-positive cells showing multinucleated morphologies (G). scale bar: 50 mm. (H, I) RepID-deficient K562 cells are accelerated to the megakaryocytic differentiation by PMA treatment. Representative images of Wright-Giemsa stained in PMA-treated K562 RepID WT and KO cells (H). scale bar: 20 mm. Transcripts of megakaryocyte marker (CD41, CD61 and DAB2) or multinucleated ratios in PMA-treated K562 RepID WT and KO cells were examined by qRT-PCR or cell counting, respectively (I). All error bars in this figure represent the standard deviation (*P-value < 0.05, **P < 0.01, ***P < 0.001, ****P < 0.0001, n. s.; not significant).

K562 cells are bipotent progenitor cells, which can be differentiated into megakaryocytes or erythroid lineages following exposure to PMA, 12-O-tetradecanoylphorbol 13-acetate (TPA), and retinoic acid (34–36). Based on these previous reports, we investigated whether RepID expression is associated with megakaryocytic differentiation. Indeed, more RepID-deficient K562 cells showed megakaryocytic morphological features, such as larger cell size with polysegmented nuclei, compared to RepID-expressing K562 cells (Fig. 2A–C). In addition, transcription levels of major megakaryocyte markers, including CD41, CD61, and DAB2, were also induced approximately 2–4 fold in RepID-deficient K562 cells compared with RepID-proficient K562 cells (Fig. 2D), suggesting that RepID may be an inhibitor of megakaryocytic differentiation.

Next, we tested whether RepID depletion could interfere with the induction of megakaryocyte markers and/or megakaryocytic differentiation. In RepID-expressing cells, DAB2 protein levels gradually increased after PMA treatment (Fig. 2E, F, WT panel). However, in RepID-depleted cells, the expression of the DAB2 protein reached maximum levels earlier than in RepID-proficient cells (48 hr in KO cells *vs.* 96 hr in WT cells, Fig. 2E, F, KO panel). These results were consistent with the confocal microscopy observations, in which DAB2 clearly appeared in polynucleated cells in response to PMA treatment and was more strongly induced in RepID-deficient cells (Fig. 2G, PMA panel). Moreover, results showing similar intensity of DAB2 levels between larger cells with polynucleate nuclei in RepID-deficient cells and single-nuclei cells in the absence of PMA exposure confirmed that DAB2 was relatively more abundant in RepID-depleted cells (Fig. 2G, CTL panel). In line with DAB2 induction, RepID-deficient K562 cells showed more advanced morphological changes, regarded as megakaryocytic differentiation (Fig. 2H), together with rapidly reaching maximum transcription levels of megakaryocyte markers, compared to RepID-expressing K562 cells (Fig. 2I). These results demonstrate that RepID-deficient K562 cells exhibit accelerated megakaryocytic differentiation.

### RepID-CRL4A dissociates from chromatin during megakaryocytic differentiation

**Figure 3:**
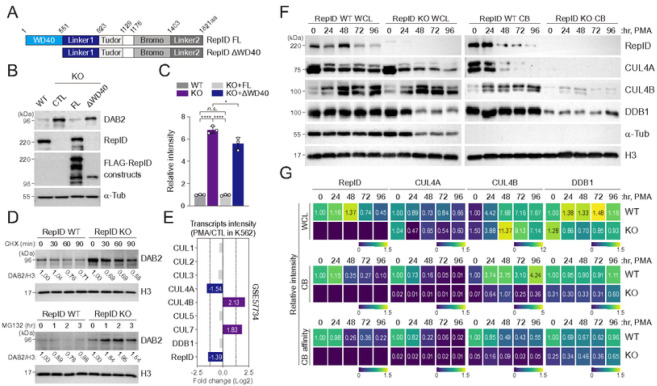
RepID-CUL4A dissociates from the chromatin during megakaryocytic differentiation (A-C) RepID WD40 domain is required for inhibition of DAB2 expression. Construction of RepID full-length (FL) and lacking WD40 fragments (ΔWD40) (A). Total protein was extracted from RepID-deficient K562 cells transfected with RepID fragments and K562 RepID WT cells as indicated, and immunoblot analysis was performed using DAB2 antibodies. Alpha-tubulin was used as a loading control (B). Quantification of DAB2 levels, normalized to its levels in RepID WT cells. All error bars represent the standard deviation from 3 independent experiments (*P-value < 0.05, ****P < 0.0001, n. s.; not significant)(C). (D) Stability of DAB2 protein is not altered regardless of RepID expression. K562 RepID WT or KO cells incubated with or without the protein synthesis inhibitor cycloheximide or proteasome inhibitor MG132. Cell lysates were subjected to the immunoblot analysis and relative densitometry was indicated. (E) Differential changes of transcription level for selected CRL components (CUL1, CUL2, CUL3, CUL4A, CUL4B, CUL5, CUL7, DDB1, RepID) in PMA-treated K562 cells (20 nM, 2 days) in published microarray dataset (GSE57734). (F, G) RepID and CUL4A dissociates from chromatin during megakaryocytic differentiation. K562 RepID WT or KO cells incubated with PMA were collected at the indicated times, and chromatin-bound fractions were extracted and immunoblot analysis was performed using anti-RepID, anti-CUL4A, anti-CUL4B and anti-DDB1 antibodies. Alpha-tubulin and histone H3 was used for loading as well as fractionation control (F). (G) Quantification of RepID, CUL4A, CUL4B and DDB1 levels in whole cell lysates and chromatin-bound fractions after normalizing alpha-tubulin or histone H3, respectively. Chromatin binding affinity was calculated by dividing the normalized chromatin-bound fractions to normalized whole cell lysates. All quantifications were presented as heatmap plots. The numbers are fold changes relative to the corresponding protein in WT cells without PMA treatment (0 hr).

Since our observations suggest that RepID may be a crucial factor for DAB2 expression, we sought to identify the mechanism of action of RepID for altering DAB2 transcription. RepID contains three functional domains: a WD40-repeat containing domain that serves as scaffold for protein-protein interactions, the two bromodomain and a tudor chromatin binding domains (24). Because we previously show that RepID WD40 domain was crucial for its functional activity during DNA replication (16), we generated stable K562 cell lines expressing FLAG-tagged full length RepID and a RepID fragment lacking the WD40 domain in RepID-depleted cells (Fig. 3A). The results showed that DAB2 expression was compromised with the RepID full-length (FL), but not with the RepID fragment lacking the WD40 domain (ΔWD40)(Fig. 3B, C). Additionally, there were no substantial alterations in the protein levels of DAB2 in both RepID-expressing and RepID-depleted cells treated with the protein synthesis inhibitor cycloheximide or the proteasome inhibitor MG132. This result suggests that DAB2 is not a substrate for CRL4, but the WD40 domain of RepID is crucial for the transcriptional repression of DAB2 (Fig. 3D).

Together with RepID, CRL4 is also a possible factor that alters gene transcription by regulating heterochromatin formation (28–30). Therefore, we investigated whether the expression or chromatin-binding ability of RepID or CRL4 could be altered during megakaryocytic differentiation. Transcriptional intensity from published microarray datasets comparing treated (20 nM, 2 days) and untreated PMA K562 cells showed a reduction in CUL4A and RepID expression but a markedly increased of CUL4B and CUL7 transcripts, whereas CRL4 adaptor DDB1 was not changed (Fig. 3E). Consistent with these observations, protein expression levels of RepID, CUL4A or CUL4B changed slightly during PMA-mediated differentiation (Fig. 3F, G, WCL panel). Interestingly, chromatin-bound RepID decreased substantially in differentiating cells, leading to CUL4A dissociation from chromatin (Fig. 3F, G, RepID and CUL4A panel). In contrast, the chromatin binding of CUL4B and DDB1 increased slightly, even in RepID-depleted cells, suggesting that these proteins may be loaded onto chromatin by another mechanism (Fig. 3F, G, CUL4B and DDB1 panel). These results also suggest that CUL4A and CUL4B may have different functions in megakaryocytic differentiation and that RepID-CRL4A dissociation from chromatin seems to be correlated with the induction of DAB2 transcription.

### DAB2 promoter forms euchromatin by dissociation of the CRL4A-JARID1A complex during megakaryocytic differentiation

**Figure 4:**
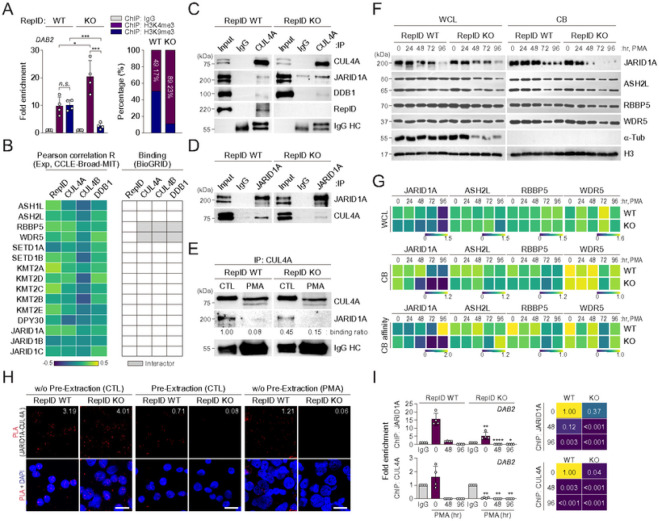
DAB2 promoter forms euchromatin by dissociation of CRL4A-JARID1A complex during megakaryocytic differentiation (A) Chromatin configuration of DAB2 promoter in RepID-deficient K562 cells. ChIP-qPCR was performed using antibodies against H3K4me3 and H3K9me3 in K562 RepID WT or KO cells to examine epigenetic configuration of the DAB2 promoter region. Data are represented as the fold enrichment compared to the IgG control after normalizing with input value. (B) Correlation between CRL4 components (RepID, CUL4A, CUL4B and DDB1) and histone H3K4 modifiers including methyltransferase and demethylase in all cancer dataset based on CCLE database using CellMinerCDB. Pearson correlation R value was presented as heatmap (left panel). Published interactions between CRL4 components and H3K4 modifiers were analyzed using public data from BioGRID database (right panel). (C, D) JARID1A interacts with CUL4A. A pull-down assay using a CUL4A antibody was performed using whole cell extracts from K562 RepID WT and KO cells and co-precipitated JARID1A, DDB1 and RepID were analyzed by immunoblotting (C). Immunoprecipitation using JARID1A antibodies to pull down CUL4A (D). (E) CUL4A dissociates from JARID1A during megakaryocytic differentiation. Whole cell extracts from K562 RepID WT and KO cells induced to megakaryocytic differentiation using PMA for 48 hours were immunoprecipitated using CUL4A antibodies, and co-precipitated JARID1A was analyzed by immunoblotting and densitometry. (F, G) Chromatin binding of JARID1A is highly decreased during megakaryocytic differentiation. The whole cell extracts and chromatin-bound fractions from K562 RepID WT or KO cells incubated with PMA as the indicated times were analyzed by immunoblot analysis using anti-JARID1A, anti-ASH2L, anti-RBBP5 and anti-WDR5 antibodies. Alpha-tubulin and histone H3 was used as the control for loading and fractionation (F). Quantification of JARID1A, ASH2L, RBBP5 and WDR5 levels in whole cell lysates and chromatin-bound fractions after normalizing alpha-tubulin or histone H3, respectively. Chromatin binding affinity was calculated by dividing the normalized chromatin-bound fractions to normalized whole cell lysates, and all quantification was presented as a heatmap plot (G). (H) Proximity ligation assay for protein interaction between CUL4A and JARID1A in with/without PMA treatment in pre- or no preextraction condition. Each red spot represents a single protein-protein interaction and DNA was stained with DAPI. scale bar: 20 mm. Red signal numbers in each cell were quantified by analyzing 315–747 cells randomly, and average numbers for positive red signals per cells are indicated. (I) JARID1A-CUL4A complex dissociates from DAB2 promoter. ChIP-qPCR of DAB2 promoter region was performed using antibodies against JARID1A and CUL4A in K562 RepID WT or KO cells treated with PMA to induce megakaryocytic differentiation. Data are presented as the fold enrichment compared to the IgG control after normalizing with input value (left panel). Relative DAB2 promoter binding ratio was indicated as heatmap compared to untreated RepID WT (right panel).

A ChIP-PCR analysis was used to determine if RepID-CRL4A influences epigenetic changes in the DAB2 promoter. The ratio of H3K4me3 to H3K9me3 clearly differed according to RepID expression, showing euchromatin formation in DAB2 promoter regions mediated by a decrease in H3K9me3 and an increase in H3K4me3 levels in RepID-depleted K562 cells (Fig. 4A). Therefore, we analyzed whether the expression of histone modifiers, including H3K4 methyltransferase and demethylase, was altered by the expression of CRL4 components, including RepID, CUL4A, CUL4B, and DDB1. However, our in-silico analyses of cancer cell lines from the CCLE of the Cellminer dataset did not show considerable expression correlations between the modifiers and CRL4 components (Fig. 4B, left panel). Next, we examined whether euchromatin formations of DAB2 promoter was due to interaction between the modifiers and CRL4 components. Although the protein-protein interaction database in BioGRID showed few modifiers including RBBP5 or WDR5 as CRL4 interactors (Fig. 4B, right panel), our immunoprecipitation analysis with antibodies directed against endogenous CUL4A or JARID1A found that CRL4A forms a complex with JARID1A (Fig. 4C, D), and their interaction decreased during megakaryocytic differentiation (Fig. 4E). In line with RepID-CRL4A dissociation from the chromatin, JARID1A chromatin binding and expression levels were also substantially reduced in response to PMA-mediated differentiation. Unlike the other modifiers we tested, high amounts of JARID1A left chromatin in RepID-deficient cells compared to RepID-expressing cells (Fig. 4F, G). To validate the interaction between CRL4A and JARID1A, we performed a proximity ligation assay (PLA) that allows *in situ* detection of protein interactions. Without pre-extraction, we did not detect substantial differences in interaction signals between RepID-expressing and RepID-depleted cells (Fig. 4H, left panel), whereas the signals for JARID1A-CRL4A interaction completely disappeared in pre-extracted RepID-deficient nuclei (Fig. 4H, middle panel), suggesting that most CRL4AJARID1A interactions may occur in soluble nuclear fractions and then be recruited to chromatin mediated by RepID. However, similar amounts of chromatin-bound JARID1A in RepID-expressing and RepID-deficient K562 cells without PMA treatment suggest that a subset of JARID1A forms a complex with CRL4A. Compared to proliferating K562 cells, cells differentiating into megakaryocytes lost the CRL4AJARID1A interaction faster in RepID-deficient cells than in RepID-proficient cells (Fig. 4H, right panel), suggesting that CRL4A dissociates from JARID1A during megakaryocytic differentiation. These results were consistent with the ChIP-qPCR results, in which the fraction of JARID1A and CRL4A bound to the DAB2 promoter decreased substantially in response to PMA treatment in both cell types (Fig. 4I). In addition, DAB2 promoter-bound disappearance of JARID1A-CRL4A was faster in RepID-depleted cells. This observation could be correlated to the fact that these cells show very low amounts of CRL4A on the DAB2 promoter (Fig. 4I). Our observations also noted that a small fraction of JARID1A can still bind to the DAB2 promoter in RepID-depleted cells and would suggest that a small fraction of JARID1A can be loaded on the DAB2 promoter in a RepID-independent manner (Fig. 4I). Taken together, our observations suggest that RepID may modulate megakaryocytic differentiation by regulating the loading of a subset of CRL4A-JARID1A onto the DAB2 promoter.

## Discussion

Megakaryocytes (MKs) are highly specialized large cells that are crucial precursors of blood platelets, small anucleate cell fragments with crucial roles in wound healing, inflammation, angiogenesis and hemostasis. MKs differentiation leads to polyploidy by endomitosis followed by a maturation process for platelet production, and these processes are governed by intensive changes in gene expression patterns. The data presented here demonstrate that megakaryocytic differentiation requires RepID dissociation from chromatin. The loss of chromatin-bound RepID compromises the loading of the CRL4A-JARID1A complex at the DAB2 promoter, leading to the initiation of DAB2 transcription, a key regulator of platelet signaling. MK differentiation was partially executed in K562 RepID-deficient cells, even in the absence of a differentiation signal, while induced MK differentiation by PMA led to faster megakaryocytes-like cell formation when using K562 RepID-depleted cells. Altogether, these observations identified the RepID-CRL4A-JARID1A complex as a putative factor in the maintenance of K562 proliferation.

RepID, known as the replication origin binding protein as well as a structural receptor for CRL4 ubiquitin E3 ligase, initiates a subpopulation of DNA replication origins and prevents over-replication via CRL4 recruitment on chromatin to ubiquitinate CDT1 (16, 17, 37–39). RepID shows direct chromatin binding ability using its three histone-code reader bromodomain, cryptic Tudor domain and WD40 domains. Genome-wide ChIP-Seq results reveal that most of the RepID binding sites are concentrated in promoter regions (16, 22, 24, 40, 41). Moreover, reduced heterochromatin formation due to compromised interaction between CRL4A and the epigenetic silencing factors EED, a subunit of the PRC2 methyltransferase, or the histone H3K4me3 demethylase JARID1C suggest that RepID-CRL4 may regulate both DNA replication and transcription landscapes (28, 42). We identified JARID1A as a novel interaction protein of CRL4A, with the JARID1A-CRL4 complex being prominently present in the soluble nuclear fraction. The JARID1A-CRL4 complex seems to be recruited to the DAB2 promoter in a RepID dependent manner to repress DAB2 transcription. Upon PMA-induced megakaryocytic differentiation of K562, we observed that the CRL4A-JARID1A complex dissociates from chromatin genome wide, including at the DAB2 promoter. This dissociation might reflect the absence of chromatin recruitment by RepID. RepID is a crucial player for cell proliferation in driver-negative tumors, including melanoma, breast, and lung cancers which lose HER2, EGFR, ALK, or BRAF expression (43). The observations reported here suggest that RepID may also play a role in hematopoietic proliferation by inhibiting CRL4A-JARID1A-mediated repression of genes correlated with megakaryocytic differentiation. For example, megakaryocytic differentiation is closely related to the canonical Wnt signaling pathway, which plays an essential role in cell fate determination, differentiation, and proliferation. Previous reports have shown that platelet function is negatively regulated by canonical Wnt signaling (44). Interestingly, RepID-repressed DAB2 is a negative regulator of Wnt/b-catenin pathway. DAB2 induction has also been reported during erythrocyte differentiation of mouse embryonic stem cells and K562 cells (45), suggesting that DAB2 may be an initial factor for cell fate decisions. These studies can lead to a better understanding of RepID’s role in erythropoiesis.

Our results revealed that the dynamics of chromatin loading differ between CUL4A (decreased loading) and CUL4B (increased loading) during induced megakaryocytic differentiation. Human CUL4A and CUL4B share approximately 82% sequence identity, 90% homology and contribute to redundant functions in cell cycle progression and embryonic development by mediating the ubiquitination of CRL4 targets (16, 46–48). However, distinctive functions of CUL4B have also been revealed in the assembly of atypical CRL4B ubiquitin ligase complexes integrated with dioxin receptors to target sex steroid receptors for degradation (49). Specific CUL4B mutations have been identified as genetic defects responsible for X-linked mental retardation (50, 51). Therefore, in line with previous reports, we propose that CUL4A and CUL4B may have specific functions on chromatin leading to the alterations of undiscovered targets and/or gene expression, that, in turn, would modulate proliferation and differentiation. It would therefore be interesting to determine which genes or proteins are differentially regulated by CRL4A or CRL4B during megakaryocytic differentiation.

**Figure 5:**
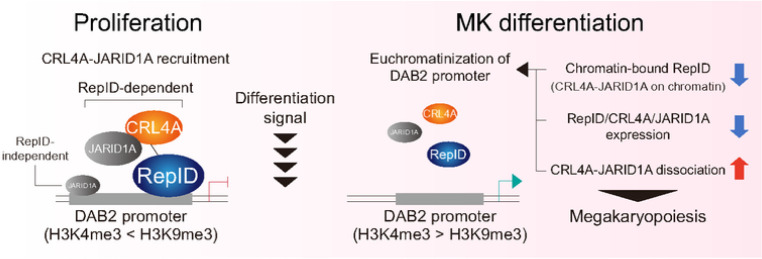
Schematic model In RepID WT cells, CRL4A forms complex with subset of JARID1A in nucleus and CRL4A-JARID1A complex is recruited to DAB2 promoter mediated by interaction between RepID WD40 domain and CRL4A. JARID1A loading on DAB2 promoter maintains its epigenetic configuration as heterochromatin status, leading to transcription repression. During megakaryocytic differentiation, CRL4A-JARID1A dissociates from the DAB2 promoter due to the reduction of chromatin binding affinity of RepID, followed by dissociation between CRL4A and JARID1A as well as between RepID and CRL4A, that leads to the increase of DAB2 transcription. In RepID depleted (RepID KO) cells, CRL4A also forms complex with subset of JARID1A in nucleus, but recruitment of this complex on DAB2 promoter is compromised due to absence of RepID. Thus, in RepID KO cells, DAB2 transcription is relatively higher than RepID WT cells, and megakaryocytic differentiation is more accelerated in response to PMA treatment.

We propose the following model for the role of RepID in repressing the expression of genes required for megakaryocytic differentiation by recruiting the CRL4A-JARID1A complex (Fig. 5). In proliferating condition, CRL4A forms a complex with JARID1A in the nucleus, that is recruited to the DAB2 promoter by RepID, that, in turn, leads to heterochromatization of DAB2 promoter and DAB2 reduced expression. A subset of JARID1A is also recruited to the DAB2 promoter in a RepID-independent manner. In response to induced megakaryocytic differentiation, CRL4A-JARID1A dissociates from the DAB2 promoter due to the three mechanisms; 1. the reduction in chromatin-bound RepID, 2. downregulation of CRL4A, JARID1A and RepID expression, 3. complex dissociation between CRL4A and JARID1A. RepID-independent JARID1A is also dissociated from the DAB2 promoter, leading to a high ratio of H3K4me3 required for the upregulation of DAB2 transcription. In RepID-deficient cells, a subset of JARID1A that interacts with CRL4A cannot be loaded onto the DAB2 promoter because CRL4A loading onto chromatin is compromised. Therefore, genes required for megakaryocytic differentiation may be partially transcribed that is translated to increased number of RepID-depleted cells with megakaryocytic traits of with an accelerated response to PMA treatment. These findings provide novel mechanistic insights into the dynamics of RepID-mediated complex-based megakaryocytic differentiation, which can form the basis for the development of new therapeutic approaches to induce platelet production.

## Data Availability

All data are contained within the article and supporting information. The datasets and analyzed data during this study are available from the corresponding author on reasonable request with permission from the Ethics Committee of the Chungbuk National University.
